# The Impact of the COVID-19 Pandemic on End-of-Life Prescribing in Ontario Nursing Homes.

**DOI:** 10.23889/ijpds.v7i3.2064

**Published:** 2022-08-25

**Authors:** Christina Milan, Colleen Webber, Anna Clarke, Sarina Isenberg, James Downar, Daniel Kobewka, Amy Hsu, Jenny Lau, Aynharan Sinnarajah, Jessica Simon, Kaitlyn Boese, Amit Arya, Rhiannon Roberts, Luke Turcotte, Michelle Howard, Colleen Maxwell, Benoit Robert, Peter Tanuseputro

**Affiliations:** 1 London School of Economics; 2 Ottawa Hospital Research Institute; 3 ICES; 4 University Health Network; 5 Alberta Health Services; 6 North York General Hospital; 7 Canadian Institute for Health Information; 8 McMaster University; 9 University of Waterloo; 10 Perley Health

## Objectives

Our preliminary work revealed significant variations in the prescribing of end-of-life symptom management medications in nursing homes prior to the onset of the COVID-19 pandemic. In this study, we sought to explore whether the prescribing of end-of-life medications in nursing homes changed with the onset of the pandemic.

## Approach

The paper utilises the first wave of the newly linked Growing up in England dataset, which consists of administrative education data for children and young people in schools and further education institutions in England linked to their 2011 Census records. It focuses on qualifications studied as well as attainment achieved of a cohorts of Gypsy, Traveller and Roma young people between age 15 (around the time of 2011 Census) and 19. The analysis explores the relationship between educational pathways and attainment for these young people and their household/family characteristics recorded in the 2011 Census.

## Results

Nursing homes in the lowest prescribing quintile prescribed, on average, 11.5% fewer end-of-life symptom management medications during COVID-19 compared to pre-pandemic. Conversely, homes in the highest quintile prescribed an average of 13.7% more medications during COVID-19. Nursing homes in the lowest quintile had more COVID-19-positive residents (33% of residents) compared to homes in the highest quintile (9% of residents). Additionally, nursing homes in the lowest prescribing quintile spent more time with active COVID-19 outbreaks compared to homes in the highest quintile (mean 72.7 days versus 24.1 days, respectively, standardized difference 0.819).

## Conclusion

The COVID-19 pandemic has disproportionately impacted nursing homes across Canada. Our findings suggest that nursing homes with low rates of prescribing of end-of-life medications prior to the pandemic had even lower prescribing rates during the pandemic. These homes were also harder hit by COVID-19 infections and outbreaks.

